# Derlin-1 Deficiency Is Embryonic Lethal, Derlin-3 Deficiency Appears Normal, and Herp Deficiency Is Intolerant to Glucose Load and Ischemia in Mice

**DOI:** 10.1371/journal.pone.0034298

**Published:** 2012-03-29

**Authors:** Yuka Eura, Hiroji Yanamoto, Yuji Arai, Tomohiko Okuda, Toshiyuki Miyata, Koichi Kokame

**Affiliations:** 1 Department of Molecular Pathogenesis, National Cerebral and Cardiovascular Center, Suita, Osaka, Japan; 2 Laboratory of Neurology and Neurosurgery, National Cerebral and Cardiovascular Center, Suita, Osaka, Japan; 3 Department of Bioscience and Genetics, National Cerebral and Cardiovascular Center, Suita, Osaka, Japan; University of Pecs Medical School, Hungary

## Abstract

Accumulation of unfolded or misfolded proteins in the endoplasmic reticulum (ER) causes a cellular condition called ER stress. To overcome ER stress, unfolded proteins are eliminated by an ER-associated degradation (ERAD) system. To explore the physiological requirements for ERAD-related membrane proteins in mammals, we generated Derlin-1–, Derlin-3–, and Herp-deficient mice by gene targeting. Complete loss of Derlin-1 caused embryonic lethality at around E7–E8 (early somite stages). In contrast, Derlin-3– and Herp-deficient mice were born alive with the expected Mendelian frequency, and were superficially indistinguishable from wild-type mice. However, in the Derlin-3– and Herp-deficient mouse organs, the expression levels of ERAD-related proteins were affected under both normal and ER stress conditions; specific effects differed among the organs. Degradation of ERAD substrates was reduced in the Herp-deficient liver, and Herp-deficient mice exhibited impaired glucose tolerance and vulnerability to brain ischemic injury, both of which are known to be implicated in ER stress. Our findings indicate that ERAD or uncharacterized functions involving Derlin-1 are essential in early embryonic development. Derlin-3– and Herp-deficient mice may become useful model animals for investigations of the physiological contribution of ERAD under stressful or pathological conditions.

## Introduction

Unfolded or misfolded proteins accumulated in the endoplasmic reticulum (ER) can induce ER stress, which leads to defects in protein homeostasis. To overcome ER stress, the cells operate several survival systems, one of which is a disposal mechanism called ER-associated degradation (ERAD). In ERAD, misfolded proteins are recognized in the ER and retrotranslocated to the cytosol to be degraded by the ubiquitin-proteasome system [1], [2], [3], [4]. Multiple proteins participate in the process; in particular, ER-membrane proteins including HRD1, Derlin, and Herp work together in functional complexes termed ERAD complexes.

HRD1 is a transmembrane E3 ubiquitin ligase that mediates ubiquitination of ERAD substrates. Yeast Hrd1p/Der3p is essential for degradation of HMG-CoA reductase and mutated carboxypeptidase Y (CPY*) [5], [6]. A protein complex consisting of Hrd1p, Hrd3p, Der1p, and Usa1p deals with ER-luminal misfolded proteins; Hrd1p is the central membrane component of this complex [7]. Mammalian HRD1 also functions in ERAD, forming a complex with other proteins [8], [9], [10].

Mammalian Derlin family proteins, Derlin-1, Derlin-2, and Derlin-3, are orthologs of yeast Der1p, which was identified as a gene that complemented a yeast mutant that cannot degrade CPY* [11]. All Derlin family members contain transmembrane domains and are embedded in the ER membrane [12], [13]. In human, Derlin-1 and Derlin-2 mRNAs were expressed ubiquitously, and expression of Derlin-3 mRNA was relatively restricted to several tissues including placenta, pancreas, spleen, and small intestine [13]. Previous reports have delineated their functional roles in ERAD. Derlin-1 is the prime candidate for the ERAD retrotranslocation [12], [14]: anti-Derlin-1 antibody inhibited retrotranslocation of ERAD substrates in an *in vitro* retrotranslocation assay [15], and overexpression of a dominant-negative Derlin-1 mutant inhibits ERAD [12]. Overexpression of Derlin-2 or Derlin-3 accelerated ERAD of misfolded glycoproteins, and knockdown of either gene inhibited the degradation [13].

Herp was first reported as an ER stress–responsive protein expressed in cultured human vascular endothelial cells [16]. Herp is a representative target gene of the unfolded protein response (UPR); the induction of Herp mRNA expression following ER stress is mediated via two *cis*-acting ER stress–response elements, ERSE and ERSE-II [17]. Although Herp may play a broad range of biological roles [18], [19], [20], [21], [22], recent reports focus on its function in ERAD. Degradation of several ERAD substrates, including connexin 43 [20], CD3δ [9], [23], κLC [24], polycystin-2 [25], and α1-antitrypsin null Hong Kong (NHK) [10] is dependent on Herp. Usa1p, the yeast ortholog of Herp, may be the scaffold protein of the functional complex containing Hrd1p [26]; it is also involved in the degradation of Hrd1p itself [27].

Although growing evidence suggests that ERAD-complex components play important roles in cellular functions, little data exist regarding their systemic significance in mammals. HRD1-deficient mice die *in utero* around embryonic day 13.5, largely due to impairment of definitive erythropoiesis [28]. Derlin-2–deficient mice exhibit perinatal lethality and represented only 4% of mice at weaning [29]. To understand the physiological roles of the Derlin family members and Herp, we generated three mouse knockouts.

## Results and Discussion

### Offspring from the Cross of Heterozygous Mice

**Figure 1:**
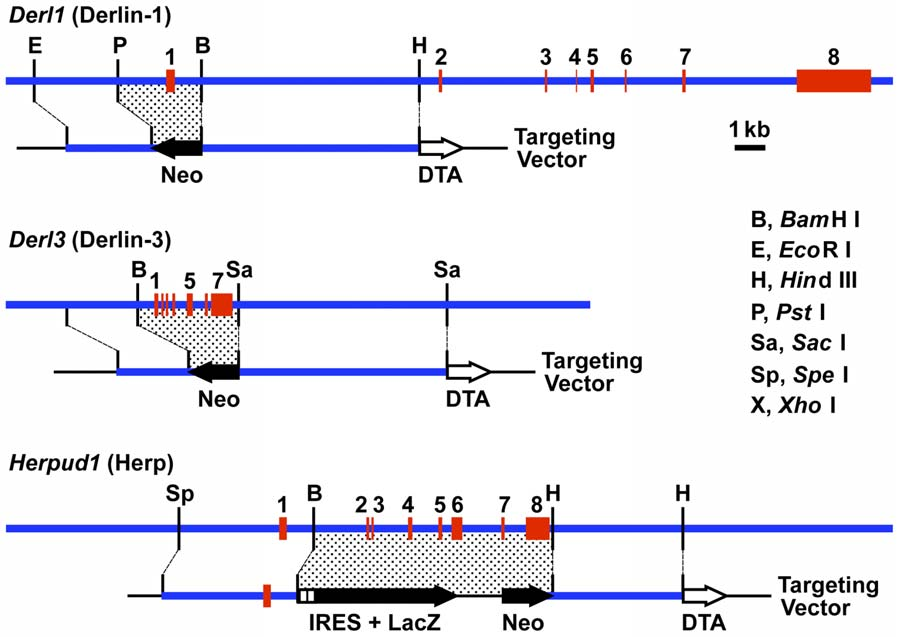
Targeted disruption of Derlin-1, Derlin-3, and Herp. Structures of the mouse *Derl1*, *Derl3*, and *Herpud1* loci, and the targeting vectors for disrupting each gene. Neo; neomycin resistance gene, IRES; internal ribosome entry site, DTA; diphtheria toxin A chain.

To generate Derlin-1–, Derlin-3–, and Herp-deficient mice, we constructed targeting vectors for disruption of the mouse *Derl1*, *Derl3*, and *Herpud1* genes (Figure 1). All the F1 heterozygous mice, *Derl1* ^+/−^, *Derl3* ^+/−^, and *Herpud1* ^+/−^, were viable and grew with no apparent phenotypic abnormalities under normal breeding conditions. Next, the heterozygous mice were crossed to generate homozygous mutants, and the genotypes of newborn offspring were determined by genomic PCR. From these matings, *Derl3* ^−/−^ and *Herpud1* ^−/−^ newborn mice were born in the expected Mendelian ratios, whereas *Derl1* ^−/−^ mice were not detected, indicating that the complete loss of Derlin-1 severely affected normal embryogenesis (Table 1).

**Table 1:** Genotype of offspring from the cross of heterozygous mice.

Cross		Genotype		*P* a
	+/+	+/−	−/−	
*Derl1* ^+/−^×*Derl1* ^+/−^	76	157	0	1.3×10^−17^
*Derl2* ^+/−^×*Derl2* ^+/−^	90	156	0	7.2×10^−19^
*Derl3* ^+/−^×*Derl3* ^+/−^	56	101	39	0.21
*Herpud1* ^+/−^×*Herpud1* ^+/−^	76	156	71	0.81

a Chi-square test.

### Early Embryonic Death of *Derl1* ^−/−^

**Figure 2:**
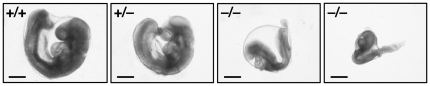
Stereomicroscopic appearance of Derlin-1–deficient mouse embryos. *Derl1* ^−/−^ embryos at E8.5 exhibited developmental delay and resorption, respectively, whereas *Derl1* ^+/+^ and *Derl1* ^+/−^ embryos were normal. Bars, 50 µm.

To identify the embryonic stages at which *Derl1* ^−/−^ mice die, we analyzed the embryos in uteri of pregnant *Derl1* ^+/−^ female mice that had been crossed with *Derl1* ^+/−^ male mice (Table 2). We collected embryos and determined their genotypes by PCR. In the *Derl1* ^+/−^ crosses, only three (2.6%) *Derl1* ^−/−^ embryos were detected at E10.5, whereas 23 *Derl1* ^+/+^ and 60 *Derl1* ^+/−^ embryos were present. At E8.5, however, the numbers of embryos, 13 (*Derl1* ^+/+^), 28 (*Derl1* ^+/−^), and 11 (*Derl1* ^−/−^), were not significantly different from the expected Mendelian frequency. This suggested that most *Derl1* ^−/−^ embryos are resorbed between E8.5 and E10.5. Indeed, by E10.5, the *Derl1* ^+/−^ crosses resulted in a high frequency (26.5%) of the embryonic resorption compared with 4.5% and 1.9% resorption in wild-type C57BL/6 and ICR, respectively (Table 2). Genotypes of the resorbed embryos were not determined, to avoid miscounting caused by commingling of maternal tissues with embryos. If resorbed embryos were assumed to be *Derl1* ^−/−^, the numbers of embryos [23 (*Derl1* ^+/+^), 60 (*Derl1* ^+/−^), and 34 (*Derl1* ^−/−^)] conformed to the expected Mendelian frequency. The appearances of embryos collected at E8.5 are shown in Figure 2. In contrast to the normal development of *Derl1* ^+/+^ and *Derl1* ^+/−^ embryos, all 11 *Derl1* ^−/−^ embryos showed obvious delay in their development; they appeared as wild-type up to E7.5. Western blotting analysis of the embryos at E7.5 demonstrated the presence of Derlin-1 in *Derl1* ^+/+^ and *Derl1* ^+/−^ and the absence in *Derl1* ^−/−^ embryos (Figure S1). In conclusion, the *Derl1* ^−/−^ mice were embryonic lethal at E7–E8.

**Table 2:** Genotype of embryos from the cross of *Derl1*+/− heterozygous mice.

Cross	Embryonic age	Number of embryos	Number of each genotype (%)				*P* a	*P* b
			+/+	+/−	−/−	Resorbed		
*Derl1* ^+/−^×*Derl1* ^+/−^	E10.5	117	23 (19.7)	60 (51.3)	3 (2.6)	31 (26.5)	1.2×10^−5^	0.34
	E8.5	55	13 (23.6)	28 (50.9)	11 (20.0)	3 (5.5)	0.79	0.97
B6^c^×B6	E10.5	67	64 (95.5)	–	–	3 (4.5)	–	–
ICR^d^×ICR	E10.5	155	152 (98.1)	–	–	3 (1.9)	–	–

The heterozygous mice were mated and the embryos in uteri were genotyped. Resorbed and rudimentary embryos were not genotyped.

a Chi-square test of +/+, +/−, and −/−, not including resorbed embryos.

b Chi-square test using +/+, +/−, and −/−, counting resorbed embryos as −/−.

c C57BL/6JJcl wild-type mice.

d Jcl:ICR wild-type mice.

In contrast, *Derl3* ^−/−^ and *Herpud1* ^−/−^ mice were born and grew normally. These findings indicated that the requirement for each Derlin in embryogenesis was different: Derlin-1 was essential in the early developmental stages (the beginning of the organogenesis), deletion of Derlin-2 results in perinatal death [29], and Derlin-3 was not essential in development. The loss of Derlin-3 may be compensated by Derlin-1 or Derlin-2. Our findings also indicated that Derlin-1, Derlin-3, and Herp are not essential for cell survival, because even embryonically lethal *Derl1* ^−/−^ survived until at least E7.5. This is consistent with the fact that Der1p, Dfm1p (Der1p homolog), and Usa1p are not essential for yeast survival.

### Effects of Derlin-3 and Herp Deficiency in Adult Mouse Organs

**Figure 3:**
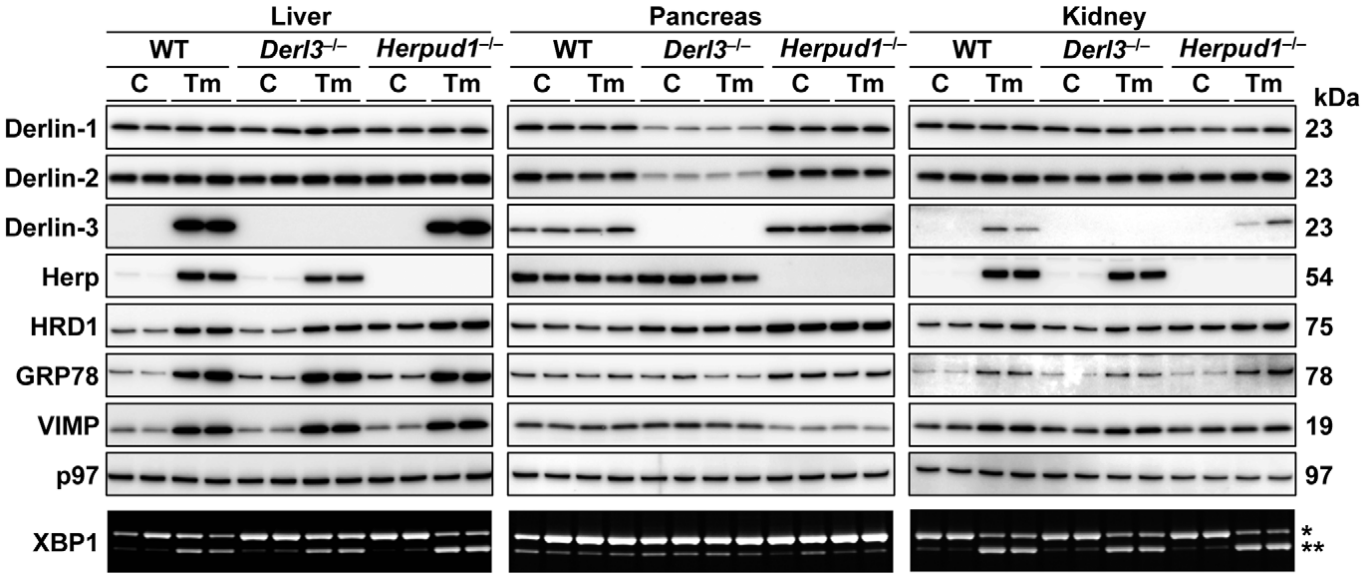
Effects of Derlin-3 and Herp deficiency on the levels of ERAD-related proteins. WT, *Derl3* ^−/−^, and *Herpud1* ^−/−^ mice were intraperitoneally injected with PBS as control (C) or Tm (2 µg/g body weight) 12 h before sacrifice. Liver, pancreas, and kidney homogenates were subjected to Western blotting using the indicated antibodies. Quantitative data are shown in Figure S2. Total RNAs prepared from the organs were subjected to RT-PCR in order to analyze the splicing of XBP1 (lowest panels). Quantitative data on the ratios (spliced/unspliced+spliced) are shown in Figure 4. *unspliced and **spliced forms of XBP1 mRNA.

**Figure 4:**
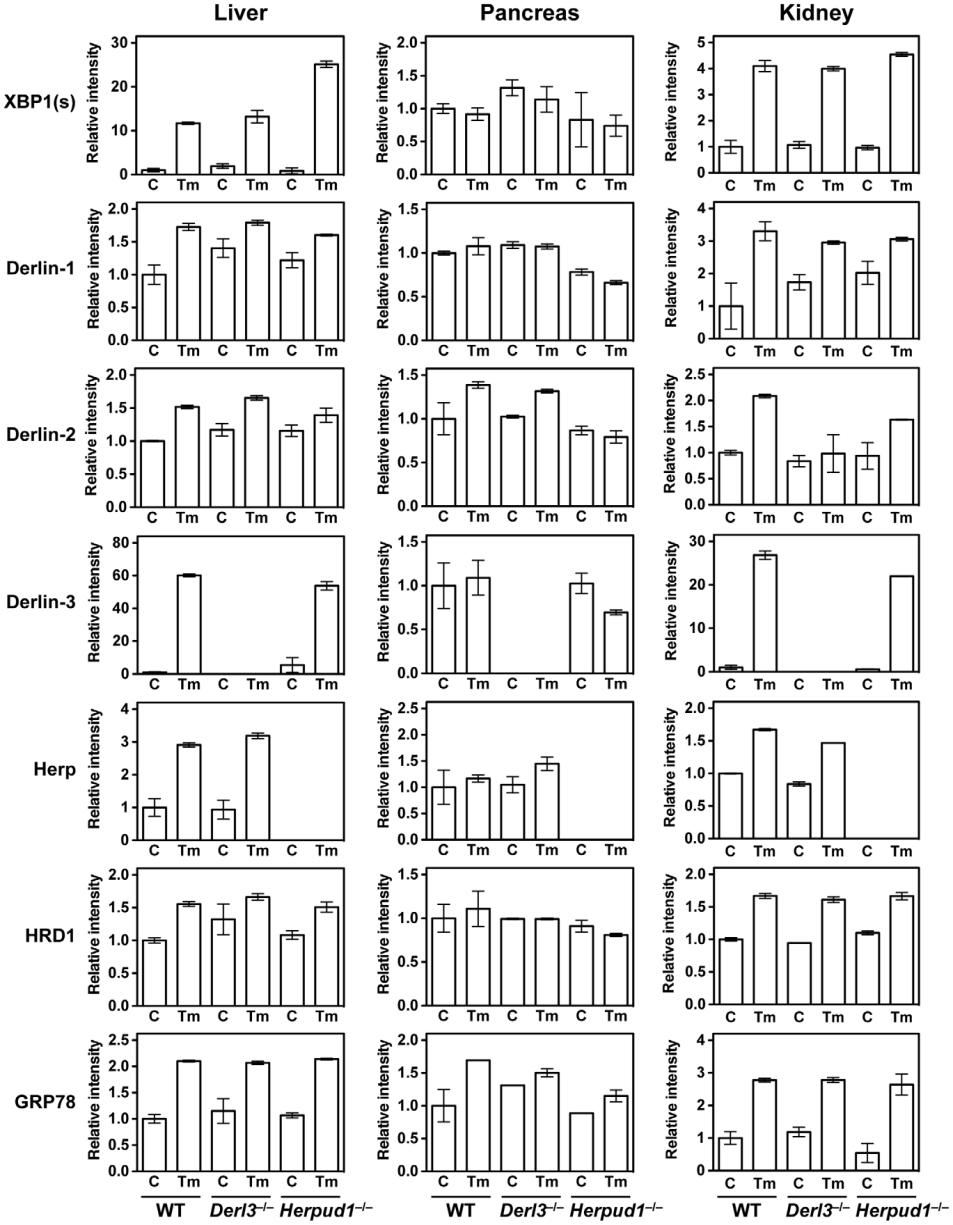
RT-PCR analysis of liver, pancreas, and kidneys from wild-type (WT), *Derl3* ^−/−^, and *Herpud1* ^−/−^ mice. Mice were intraperitoneally injected with PBS as control (C) or Tm (2 µg/g body weight) 12 h before sacrifice. Total RNAs were prepared from the liver, pancreas, and kidneys and subjected to RT-PCR. The products were analyzed by gel electrophoresis, followed by staining with SYBR Green I. Each band intensity was normalized to the mean intensity for PBS-injected WT mice. Data are expressed as means with range (*n* = 2). XBP1 (s); spliced form of XBP1 mRNA.

Although both *Derl3* ^−/−^ and *Herpud1* ^−/−^ mice were born and grew normally, deficiency of Derlin-3 or Herp may affect functionally related proteins. To investigate the effect of the knockouts on the expression levels of ERAD-related proteins, we prepared tissue homogenates and total RNA from the liver, pancreas, and kidneys of control wild type (WT), *Derl3* ^−/−^, and *Herpud1* ^−/−^ male mice that had been injected intraperitoneally 12 h before dissection with either PBS (control) or the protein N-glycosylation inhibitor tunicamycin (Tm), in order to induce pharmacological ER stress. The samples were subjected to Western blotting (Figures 3 and S2) and RT-PCR analyses (Figure 4).

In the WT liver, protein levels of Derlin-3 and Herp were quite low under normal conditions (Figure 3, left panels, C), but dramatically elevated 12 h after Tm treatment (left panels, Tm). HRD1, GRP78 (an ER-resident molecular chaperone), and VIMP (VCP-interacting membrane protein) were also increased by Tm treatment in WT mice. Similarly, in the *Derl3* ^−/−^ and *Herpud1* ^−/−^ liver, the levels of HRD1, GRP78, and VIMP were increased by Tm treatment. Although the Herp level was also increased by Tm treatment in the *Derl3* ^−/−^ liver, its levels were reduced by approximately 30% as compared with those in the Tm-treated WT (Figure S2), suggesting some physical or functional interactions between Derlin-3 and Herp. In the *Herpud1* ^−/−^ liver, regardless of Tm treatment, HRD1 level was higher than in WT. This may be partly explained by the fact that degradation of yeast Hrd1p is dependent on Usa1p (Herp ortholog) [27]. The levels of Derlin-1, Derlin-2, and AAA-ATPase p97/VCP were little affected by Derlin-3 or Herp deficiencies in the liver.

In the pancreas (Figure 3, middle panels), the levels of Derlin-1 and Derlin-2 were remarkably decreased by deficiency of Derlin-3 but not Herp. In the *Herpud1* ^−/−^ pancreas, the level of VIMP was lower than in WT and *Derl3* ^−/−^ pancreas, whereas the levels of Derlin-3, HRD1, and GRP78 were higher. In all three mouse strains, Tm had very little effect on expression levels of ERAD-related proteins in the pancreas, in contrast to the situation in the liver and kidneys. Although Derlin-3 was almost absent in the liver (left panels) and kidneys (right panels) under normal conditions, Derlin-3 could be clearly detected in the pancreas even without Tm treatment (middle panels). In the kidneys (right panels), responses to Tm treatment were broadly similar to those in the liver (left panels), but Tm-dependent upregulation of Derlin-3, GRP78, and VIMP in the kidneys was moderate compared to the liver. Thus, these findings indicated that the responses to pharmacologically induced ER stress vary among different organs.

The differences among organs in the effects of Tm and genetic deficiencies were observed not only at the protein level but also the mRNA level: the splicing of XBP1 mRNA, an indicator of the activation of ER stress sensor IRE1 [30], was induced by Tm treatment in the liver and kidneys, but not in the pancreas, of all the three mouse strains (Figures 3 and 4). The mRNA levels of other ERAD-related proteins were also investigated by RT-PCR (Figure 4): some of them (e.g., Derlin-3 and Herp mRNAs) exhibited dynamic changes similar to those observed in their protein levels, in all three organs; others (e.g., Derlin-1, Derlin-2, and HRD1 mRNAs) exhibited changes distinct from those observed in their protein levels. The decrease in the Derlin-1 and Derlin-2 protein levels in the *Derl3* ^−/−^ pancreas was likely post-translational because their mRNA levels were not decreased by Derlin-3 deficiency (compare Figures S2 and 4). The increased protein levels of HRD1 in the *Herpud1* ^−/−^ pancreas and kidney may indicate its resistance to Herp-dependent degradation as is the case in the liver. The inconsistency in the protein and mRNA levels of ERAD-related factors may be reflective of the complex, systemic, and organ-specific regulation of each protein at the level of transcription, translation, co-/post-translational modification, and degradation.

### Effect of Herp Deficiency on ERAD Substrate Degradation

**Figure 5:**
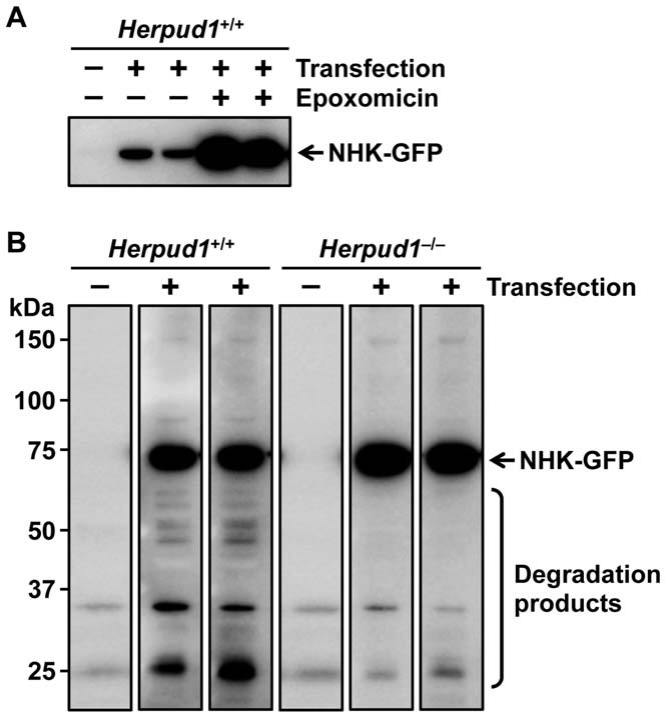
Effect of Herp deficiency on NHK-GFP degradation. (**A**) NHK-GFP was expressed in the livers of *Herpud1* ^+/+^ mice in the absence or presence of epoxomicin (*n* = 2), and detected by Western blotting using anti-GFP antibodies. (**B**) NHK-GFP was expressed in the livers of *Herpud1* ^+/+^ and *Herpud1* ^−/−^ mice (*n* = 2), and detected by Western blotting using anti-GFP antibodies.

Partial loss and quantitative imbalance in ERAD-related factors were expected to affect the function of ERAD. Then, we utilized a hydrodynamics-based *in vivo* gene transfection technique, to observe the degradation of ERAD substrates in mice. This technique provides introduction and expression of exogenous genes in whole animals, especially in the liver [31]. We injected a plasmid DNA encoding NHK with a C-terminal green fluorescent protein (GFP) tag to *Herpud1* ^+/+^ and *Herpud1* ^−/−^ mice. NHK, a truncated mutant of human α1-antitrypsin mainly produced by liver cells, and NHK-GFP are well-characterized as model substrates for ERAD [32], [33], [34], [35], [36]. Herp participates in the degradation of NHK in HeLa cells [10]. Western blotting of the *Herpud1* ^+/+^ liver expressing NHK-GFP detected the expected band of NHK-GFP, and its levels were elevated by proteasome inhibition (Figure 5A). The exogenous expression of NHK-GFP in the *Herpud1* ^+/+^ liver produced not only the intact band but also its fragment bands (Figure 5B), suggesting that NHK-GFP is degraded by ERAD in the mouse liver. In contrast, in the *Herpud1* ^−/−^ liver, the levels of the fragment bands were decreased with the accumulation of the intact band. These results indicated that Herp deficiency led to a partial defect in ERAD at least in the liver. Similarly, the other organs of *Herpud1* ^−/−^ mice and of *Derl3* ^−/−^ mice may also have some defect in ERAD. In that case, *Herpud1* ^−/−^ and *Derl3* ^−/−^ mice were expected to exhibit abnormality in challenge tests.

### Glucose Tolerance and Insulin Response

**Figure 6:**
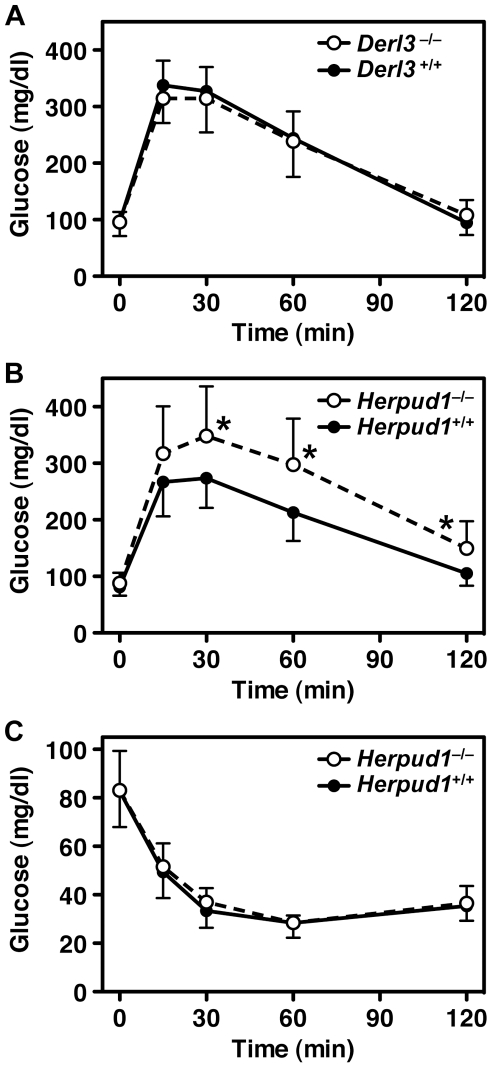
Glucose tolerance and insulin response tests. Blood glucose levels were examined at the indicated time points after intraperitoneal injection of glucose (**A** and **B**) or insulin (**C**) into *Derl3* ^−/−^ (**A**, open symbols), *Derl3* ^+/+^ (**A**, filled symbols), *Herpud1* ^−/−^ (**B** and **C**, open symbols), and *Herpud1* ^+/+^ mice (**B** and **C**, filled symbols). Data are expressed as the means with error bars of standard deviation (**A**, *n* = 12; **B**, *n* = 18; **C**, *n* = 16). Asterisks indicate *p*<0.01 (t-test) for *Herpud1* ^−/−^ vs. *Herpud1* ^+/+^ mice.

The changes in ERAD-related proteins and defect in ERAD may induce some systemic phenotypes in Derlin-3– and Herp-deficient mice, especially under stressful conditions. Then, we compared tolerance to conditions associated with ER stress between *Derl3* ^+/+^ and *Derl3* ^−/−^ and between *Herpud1* ^+/+^ and *Herpud1* ^−/−^ mice. We performed a glucose tolerance test, because blood glucose levels are a good index of vulnerability to ER stress [37], [38]. After glucose was intraperitoneally injected in fasted mice, blood glucose levels in the tails were measured over time (Figure 6). At all time points examined, blood glucose levels in *Derl3* ^−/−^ mice were quite similar to those of *Derl3* ^+/+^ mice (Figure 6A). In contrast, although blood glucose levels in *Herpud1* ^−/−^ mice were normal before glucose administration, *Herpud1* ^−/−^ mice showed significantly higher levels of blood glucose than *Herpud1* ^+/+^ mice after glucose administration (Figure 6B). Because blood glucose levels were normal in *Herpud1* ^−/−^ mice after insulin injections (Figure 6C), Herp deficiency does not appear to cause aberrant responses to insulin. These results indicated that glucose tolerance was impaired not in *Derl3* ^−/−^ mice but in *Herpud1* ^−/−^ mice. Hyperglycemia has been reported in PERK- [39] and IRE1α-deficient mice [40], both of which are UPR trigger proteins that sense ER stress. Our finding in *Herpud1* ^−/−^ mice is the first case of abnormal glucose homeostasis caused by genetic disruption of one UPR target protein contained in ERAD complexes.

### Cerebral Infarction Model

**Figure 7:**
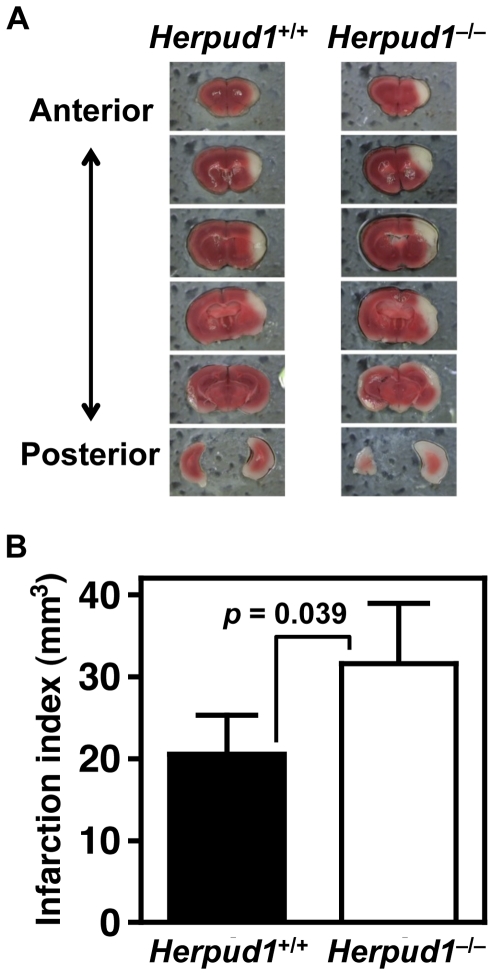
Cerebral infarction models. Neocortical infarction was induced using the temporary three-vessel occlusion method. Representative images of cerebral infarctions from *Herpud1* ^+/+^ and *Herpud1* ^−/−^ mice stained with 2,3,5-triphenyl-tetrazolium chloride 24 h after ischemia are shown (**A**). White areas indicate infarct regions. Infarction volumes (*n* = 6) are expressed as the means with error bars of standard deviation (**B**).

ER stress is induced in ischemic areas of the cerebrum, in which an infarction area forms as a result of lethal stress to neuronal cells [41], [42]. Herp contributes to protecting neural cells against ER stress-induced apoptosis *in vitro* [21], [22]. In this study, to examine the effects of Herp deficiency on resistance to cerebral ischemia *in vivo*, we measured the size of infarct lesions after three-vessel occlusion to cause temporary focal ischemia limited in the neocortex (Figure 7). Twenty-four hours after a 30-min ischemic period, infarction volumes were significantly larger in the neocortices of *Herpud1* ^−/−^ mice compared with *Herpud1* ^+/+^ mice, indicating that Herp mediates resistance to the cerebral ischemia and reperfusion, probably via increasing resistance of the ER to ischemia-derived ER stress.

In summary, our study demonstrated that Derlin-1–deficient mice were embryonically lethal at much earlier stages (E7–E8), in contrast to HRD1- and Derlin-2–deficient mice, which die at E13.5 [28] and perinatally [29], respectively. At present, it is not clear whether the embryonic death of *Derl1* ^−/−^ mice is caused by a defect in ERAD function or by a loss of some as-yet uncharacterized function of the gene. We also showed that Derlin-3– and Herp-deficient mice were born and grew normally, but exhibited some changes in ERAD-related protein components in the liver, pancreas, and kidneys. In addition, the *in vivo* challenge tests indicated that Herp plays a protective role under certain stressful conditions to the ER. These mice may be useful for investigations of the physiological contribution of ERAD under stressful or pathological conditions such as diabetes and ischemia.

## Materials and Methods

### Ethics Statement

All live mouse experiments were approved by the Animal Care and Use Committee of the National Cerebral and Cardiovascular Center (approval number: 11019), and were performed in accordance with institutional and national guidelines and regulations.

### Generation of Knockout Mice

F1 heterozygous mice, *Derl1* ^+/−^ and *Derl3* ^+/−^, were generated from C57BL/6J-derived Bruce-4 embryonic stem cells (Millipore), and crossed to wild-type C57BL/6JJcl mice (CLEA Japan) for breeding. *Herpud1* ^+/−^ mice were generated from 129/SvJ-derived Go Germline embryonic stem cells (Incyte Genomics), and backcrossed to wild-type C57BL/6CrSlc mice (Japan SLC) for 10 generations. Targeting strategies for each gene are shown in Figure 1. The genotypes of offspring were examined by PCR analysis of DNA isolated from ear biopsy. The primer sequences and product sizes are listed in Table S1.

### Analysis of Embryos

In crosses of *Derl1* ^+/−^ mice, embryos were microscopically isolated from uteri 7, 8, or 10 days after successful copulation, and the developmental stages were confirmed by appearance. The genotypes of embryos were determined by PCR as described above. To increase gestation efficiency, *Derl1* ^+/−^ mice with mixed genetic backgrounds, C57BL/6JJcl and Jcl:ICR (CLEA Japan), were used for the embryonic studies.

### Tissue Preparation

To examine the effects of pharmacologically induced ER stress *in vivo*, 18-h fasted male mice were intraperitoneally injected with PBS or tunicamycin (WAKO, 200 µg/ml dissolved in PBS) at a dose of 2 µg/g body weight. After 12 h, the anesthetized mice were perfused with PBS, and their liver, pancreas, and kidneys were excised. The organ tissues were homogenized by Polytron PT1200 (Kinematica) in SDS-PAGE sample buffer. Tissue homogenates were subjected to Western blotting. Equal loading of the samples was confirmed by quantitating protein levels in gels stained with GelCode Blue Stain Reagent (Thermo Scientific). Alternatively, for RT-PCR analysis, total RNA was prepared from the excised tissues using the RNeasy Mini Kit (Qiagen).

### Western Blotting

Anti-Derlin-3 antibody was raised in rabbits against a keyhole limpet hemocyanin–conjugated peptide, D^215^PDYLPLPEEQPEL^228^, derived from mouse Derlin-3. Anti-Herp was raised against S^122^DGLRQREVLRNLS^135^, derived from mouse Herp. These antibodies were subjected to affinity purification before use. Anti-Derlin-1 (MBL), anti-Derlin-2 (MBL), anti-HRD1 (Abgent), anti-VIMP (Sigma), anti-p97 (Santa Cruz Biotechnology), anti-KDEL (Stressgen Biotechnologies), anti-ß-actin (Sigma), and anti-GFP (Clontech) antibodies were purchased for use. Tissue homogenates were subjected to SDS-PAGE and transferred to PVDF membranes (Bio-Rad). The membranes were blocked with 5% skim milk, incubated with primary antibodies, and then incubated with HRP-labeled anti-rabbit or mouse IgG (Kirkegaard & Perry Laboratories). Signals were developed using Immobilon Western Chemiluminescent HRP Substrate (Millipore), and detected using an Image Analyzer LAS-3000 (Fujifilm).

### RT-PCR

Total RNA prepared from mouse tissues was subjected to RT-PCR using the OneStep RT-PCR Kit (Qiagen). The primer sequences and product sizes are listed in Table S1. RT-PCR for XBP1 was designed to amplify both 140-bp (unspliced form) and 114-bp (spliced form) products. The PCR products were separated by polyacrylamide gel electrophoresis and stained with SYBR Green I Nucleic Acid Gel Stain (Lonza). The bands were detected with the LAS-3000 and quantified using the Image Gauge software (Fujifilm). Band intensities were normalized against GAPDH for target genes other than XBP1. For XBP1, the ratios of the spliced form to the unspliced plus spliced forms were evaluated.

### Hydrodynamics-Based *In Vivo* Gene Transfection

The open reading frame of NHK was inserted into pAcGFP1-N1 plasmid vector (Clontech) for expression of NHK-GFP. The plasmid DNA was injected into male mice using a hydrodynamics-based transfection technique [31]. Briefly, 5 µg of the plasmid DNA was diluted in 1.5–2.2 ml (a volume equivalent to 8% of the body weight) of TransIT-EE Delivery Solution (Mirus Bio) and injected via the tail vein within 5–10 s. After 12 h, the anesthetized mice were perfused with PBS, and their liver was excised. Alternatively, the proteasome inhibitor epoxomicin (Peptide Institute, 0.2 mM dissolved in PBS) were intraperitoneally injected into mice at a dose of 15 µl/g body weight 12 h after hydrodynamics-based transfection, and after 12 h, the liver was excised. The liver tissues were homogenized by Polytron PT1200 in an ice-cold buffer (10 mM HEPES, 220 mM mannitol, 70 mM sucrose; pH 7.4) containing Complete Protease Inhibitor Cocktail (Roche), and subjected to subcellular fractionation using centrifugation. The microsomes were solubilized by a buffer containing 1% digitonin on ice, and subjected to Western blotting after removal of debris by centrifugation.

### Glucose Tolerance and Insulin Response Tests

Glucose (200 mg/ml) was intraperitoneally injected into 13-week-old fasted male mice (2 mg/g body weight). Alternatively, human insulin (100 mU/ml, Lilly) was intraperitoneally injected into 14-week-old fasted male mice (1 mU/g body weight). Before the injection and 15, 30, 60, and 120 min postinjection, a drop of blood was collected from the tail end, and the glucose level was measured using GlucoCard GT-1810 (Arkray). All mice examined were confirmed to carry the normal *Nnt* (nicotinamide nucleotide transhydrogenase) gene by PCR-based genotyping using primer pairs, CAGGTAAGAAAGCTCCTGTTT/GTGCATTGAACCCTCAAAGG for the normal allele product (235 bp) and GTCTGATACGTCCTTCATGGT/CTAGCCTTTCAGTTTTCAGGA for the deleted allele product (344 bp). *Nnt* is occasionally disrupted by deletion of exons 7–11 in the C57BL/6J mouse substrains, which may lead to glucose intolerance and reduced insulin secretion [43], [44].

### Ischemia Stroke Model

To assess tolerance to cerebral ischemia, 9-week-old male mice were subjected to the three-vessel occlusion technique [45], [46]. Briefly, the bilateral common carotid arteries were exposed and clipped under anesthesia to cause occlusion for the desired period of time. The left middle cerebral artery (MCA) was exposed by drilling a burr hole in the skull, and cauterized at the lateral edge of the olfactory tract to induce focal ischemia for cortical neurons in the MCA region. After 30 min of the three-vessel occlusion, the clips on the common carotid arteries were removed, which effectively terminate the induction of focal ischemia by increasing the collateral cerebral blood flow toward the targeted MCA territory up to the level that normal neurons never die. After 24 h, the mice were administered an overdose of pentobarbital and intracardially perfused with ice-cold PBS. The brain was removed, cut into six 1-mm-thick slices beginning at the frontal tip, and immersed in 2% 2,3,5-triphenyl-tetrazolium chloride to stain the viable tissues. The slices were then immersed in 4% paraformaldehyde/PBS, and the infarct lesion and hemispheric areas of each slice were measured by computer-assisted image analysis system. Finally, the infarct lesion size was adjusted for edema (infarction index) and calculated for each animal.

## Supporting Information

Figure S1 **Expression of Derlin-1 in** ***Derl1*** **^+/+^,** ***Derl1*** **^+/−^, and** ***Derl1*** **^−/−^ mouse embryos.** The embryos resulting from *Derl1* ^+/−^ matings were isolated from uteri at E7.5. Approximately half of each embryo was used for DNA preparation followed by PCR-genotyping, and the other half was subjected to Western blotting using anti-Derlin-1 and anti-ß-actin antibodies.
(PDF)

Figure S2 **Western blotting analysis of liver, pancreas, and kidneys from wild-type (WT),** ***Derl3*** **^−/−^, and** ***Herpud1*** **^−/−^ mice.** Mice were intraperitoneally injected with PBS as control (C) or tunicamycin (Tm) 12 h before sacrifice. Liver, pancreas, and kidney homogenates were subjected to Western blotting using the indicated antibodies. Band intensities were normalized to the mean intensity of PBS-injected WT mice (for samples other than Derlin-3 and Herp) or Tm-injected WT mice (for Derlin-3 and Herp). Data are expressed as means with range (*n* = 2). The original blots are shown in Figure 3.
(PDF)

Table S1 **Primer sequences for genotyping PCR and RT-PCR.**
(PDF)
