# Supplementary material for: Derlin-1 Deficiency Is Embryonic Lethal, Derlin-3 Deficiency Appears Normal, and Herp Deficiency Is Intolerant to Glucose Load and Ischemia in Mice
Source: PLoS One. 2012 Mar 29;7(3):e34298. doi: 10.1371/journal.pone.0034298 (PMC3315519; doi:10.1371/journal.pone.0034298)
Supplement: Figure S1 — Expression of Derlin-1 in Derl1 +/+, Derl1 +/−, and Derl1 −/− mouse embryos. The embryos resulting from Derl1 +/− matings were isolated from uteri at E7.5. Approximately half of each embryo was used for DNA preparation followed by PCR-genotyping, and the other half was subjected to Western blotting using anti-Derlin-1 and anti-ß-actin antibodies. (PDF) [file pone.0034298.s001.pdf]

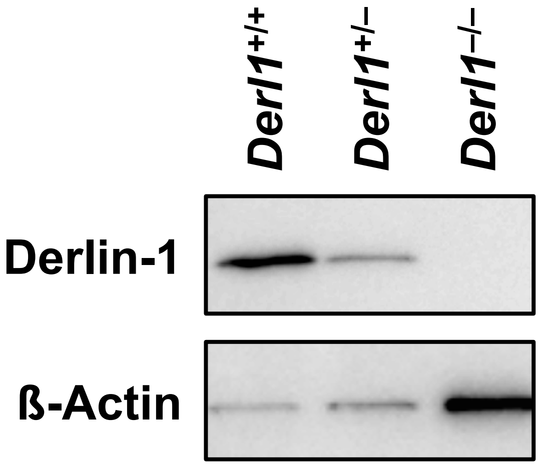

Figure S1. Expression of Derlin-1 in *Derl1*$^{+/+}$, *Derl1*$^{+/–}$, and *Derl1*$^{–/–}$ embryos at E7.5. The E7.5 embryos resulting from *Derl1*$^{+/–}$ matings were subjected to PCR-genotyping and Western blotting using anti-Derlin-1 and anti-ß-actin antibodies.
