# Supplementary material for: Derlin-1 Deficiency Is Embryonic Lethal, Derlin-3 Deficiency Appears Normal, and Herp Deficiency Is Intolerant to Glucose Load and Ischemia in Mice
Source: PLoS One. 2012 Mar 29;7(3):e34298. doi: 10.1371/journal.pone.0034298 (PMC3315519; doi:10.1371/journal.pone.0034298)
Supplement: Figure S2 — Western blotting analysis of liver, pancreas, and kidneys from wild-type (WT), Derl3 −/−, and Herpud1 −/− mice. Mice were intraperitoneally injected with PBS as control (C) or tunicamycin (Tm) 12 h before sacrifice. Liver, pancreas, and kidney homogenates were subjected to Western blotting using the indicated antibodies. Band intensities were normalized to the mean intensity of PBS-injected WT mice (for samples other than Derlin-3 and Herp) or Tm-injected WT mice (for Derlin-3 and Herp). Data are expressed as means with range (n = 2). The original blots are shown in Figure 3. (PDF) [file pone.0034298.s002.pdf]

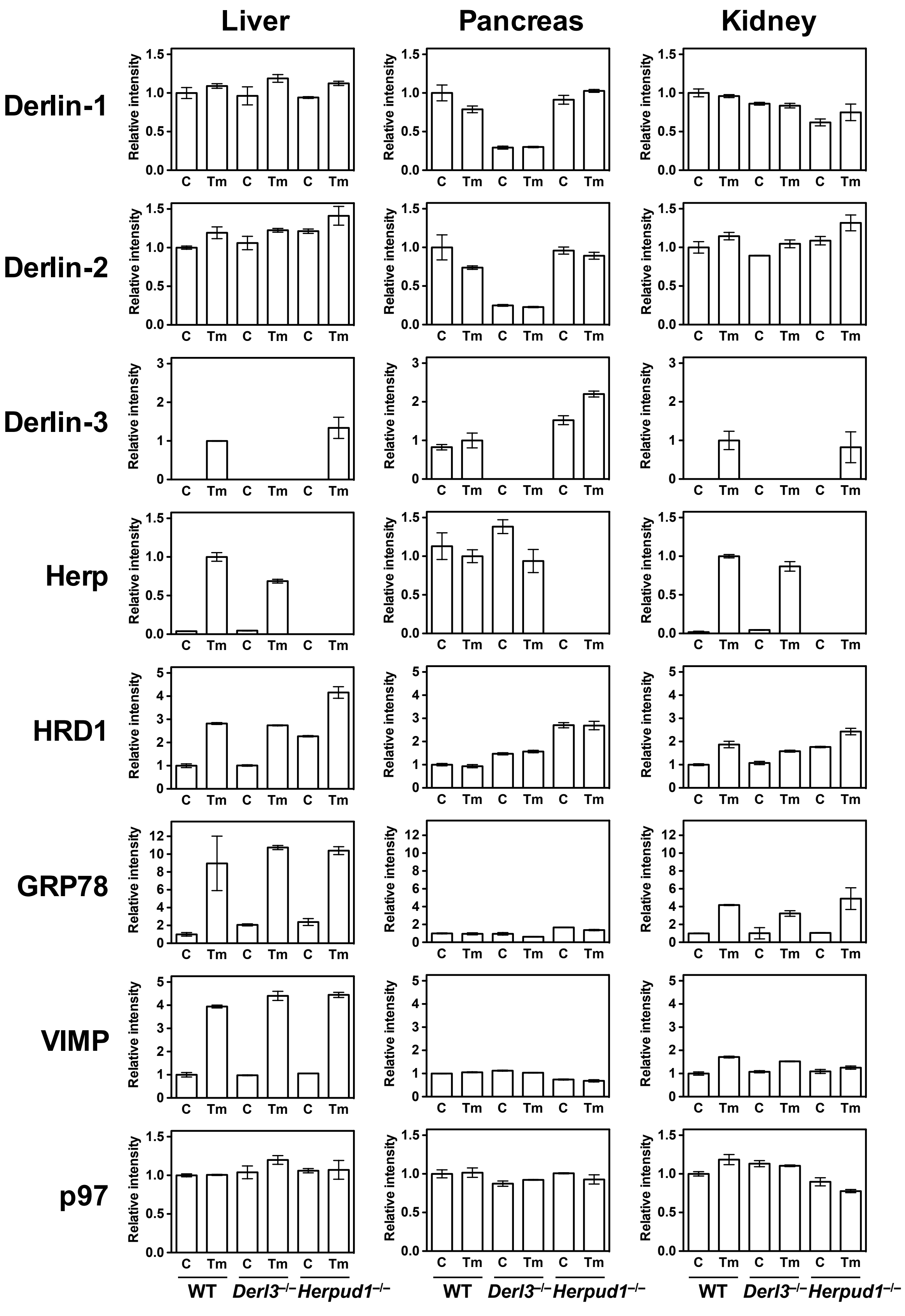

Figure S2. Western blotting analysis of liver, pancreas, and kidneys from wild-type (WT), *Derl3*$^{-/-}$, and *Herpud1*$^{-/-}$ mice. Mice were intraperitoneally injected with PBS as control (C) or tunicamycin (Tm) 12 h before sacrifice. Liver, pancreas, and kidney homogenates were subjected to Western blotting using the indicated antibodies. Band intensities were normalized to the mean intensity of PBS-injected WT mice (for samples other than Derlin-3 and Herp) or Tm-injected WT mice (for Derlin-3 and Herp). Data are expressed as means with range (n = 2). The original blots are shown in Fig. 3.
