# Supplementary material for: Derlin-1 Deficiency Is Embryonic Lethal, Derlin-3 Deficiency Appears Normal, and Herp Deficiency Is Intolerant to Glucose Load and Ischemia in Mice
Source: PLoS One. 2012 Mar 29;7(3):e34298. doi: 10.1371/journal.pone.0034298 (PMC3315519; doi:10.1371/journal.pone.0034298)
Supplement: Table S1 — Primer sequences for genotyping PCR and RT-PCR. (PDF) [file pone.0034298.s003.pdf]

Table S1. Primer sequences for genotyping PCR and RT-PCR.

Genotyping PCR

Gene	Forward	Reverse	Product (bp)
Derlin-1	ACCAGAGTTATGGGTGACTGT	TGTGCATTTCCACATACTTTC	525
Derlin-1*	ACCAGAGTTATGGGTGACTGT	CTTCCTCGTGCTTTACGGTATC	446
Derlin-3	TCTGGACCATGATAGACAGTG	GAAAAGAACCAATAGCAAGGA	393
Derlin-3*	TCTGGACCATGATAGACAGTG	CTTCCTCGTGCTTTACGGTATC	423
Herp	CCCCTCCCCCTTTGGTTGACA	TCCAGGGGCTTAGACGCTTAC	343
Herp*	CCCCTCCCCCTTTGGTTGACA	TGGACCTGGGAGTGGACACCT	252

* Targeted alleles.

RT-PCR

Gene	Forward	Reverse	Product (bp)
XBP1	GATCCTGACGAGGTTCCAGAG	AAGATGTTCTGGGGAGGTGAC	140, 114
Derlin-1	TCATGTCCCCAGAGTTGCAG	AACACACATCCGCAACGAGG	547
Derlin-2	CAACCCACTACCTGAAGAGC	CCATTTACACCACGGCCTTG	412
Derlin-3	CAAGAGACTGCTGCTGACCC	GATTCCTGTTGACTGGGCTG	367
Herp	ATCTCTAGGCCTGAGGCTGTC	GCAGTGGCAGCTAAGTATTGC	146
HRD1	CTTCCCCACCAATTCCTGAAG	AGTTCTGCAGCATCAGGCTCT	100
GRP78	TCTCAGCATCAAGCAAGGATT	CTTCATGGTAGAGCGGAACAG	120
GAPDH	AGAAACCTGCCAAGTATGATG	CTGTAGCCGTATTCATTGTCA	214
